# A Global Review on the Utility of Genetic Testing for Familial Hypercholesterolemia

**DOI:** 10.3390/jpm10020023

**Published:** 2020-04-14

**Authors:** Rachele M. Hendricks-Sturrup, Jodi Clark-LoCascio, Christine Y. Lu

**Affiliations:** 1 Department of Population Medicine, Harvard Pilgrim Health Care Institute and Harvard Medical School, Boston, MA 02215, USA; christine_lu@harvardpilgrim.org; 2 Pallavi Patel College of Health Care Sciences, Nova Southeastern University, Fort Lauderdale, FL 33314, USA; jodiclar@nova.edu

**Keywords:** hyperlipoproteinemia type II, familial hypercholesterolemia (FH), genetic testing, health policy, clinical utility

## Abstract

Familial hypercholesterolemia (FH) is a genetic disorder of cholesterol metabolism that affects an estimated 1/250 persons in the United States and abroad. FH is hallmarked by high low-density lipoprotein (LDL) cholesterol and an increased risk of premature atherosclerotic cardiovascular disease. This review summarizes recent global evidence showing the utility of FH genetic testing across diverse populations. Clinical and other qualitative outcomes following FH genetic testing were improved FH diagnosis, treatment initiation or continued treatment, treatment modification, improved total or LDL cholesterol levels, education on lifestyle management, and genetic counseling. This summary of evidence should be considered by those seeking overall evidence and knowledge gaps on the utility of FH genetic testing from a global perspective and for certain ethnic and age populations. These findings can be used to inform insurance policies and coverage decisions for FH genetic testing, policy recommendations to reduce the clinical and public health burden of FH, clinical practice and guidelines to improve the management of FH populations, and ongoing research involving FH genetic testing. We conclude that further investigations are needed to examine: (1) non-clinical outcomes following FH genetic testing; (2) patient-reported outcomes following FH genetic testing to convey patient experiences, values, and goals; and (3) clinical outcomes following FH genetic testing in non-Caucasian and pediatric populations in the United States and abroad.

## 1. Introduction

Familial hypercholesterolemia (FH) is a genetic disorder of cholesterol metabolism that is associated with very high cholesterol levels (high cholesterol defined as total blood cholesterol (TC) > 200 mg/dL or 5.2 mmoL/L; LDL cholesterol > 130 mg/dL or 3.4 mmoL/L) from birth [1,2,3]. It affects an estimated 1/250 persons in the United States (US); however, with variation among and across populations of certain ancestries or ethnicities [1,2,3]. Heterozygous gene variations inherited in a Mendelian and an autosomal dominant fashion in apolipoprotein B (ApoB), LDL receptor (LDLR), and proprotein convertase subtilisin/kexin type 9 (PCSK9) account for nearly 70–95% of FH cases [4,5,6,7]. Variations in signal-transducing adaptor protein 1 (STAP1) and apolipoprotein E (ApoE) are also associated with FH [8,9,10]. Variations in LDLR adaptor protein 1 (LDLRAP1) lead to autosomal recessive FH [11]. 

An individual with FH may experience various symptoms depending on the associated genotype. Heterozygous individuals can display typical cardiovascular disease symptoms, which include arterial plaques (coronary arteries and proximal aorta) and premature atherosclerotic cardiovascular disease (age <55 years in males; <65 years in females) [12]. Homozygous individuals can display additional symptoms that include xanthelasma and/or xanthoma [7,13]. Severe coronary artery disease (CAD) symptoms can manifest and include ischemic cardiomyopathy and highly premature fatal and non-fatal myocardial infarction, angina, and stroke [13,14]. 

An International Classification of Diseases, Tenth Revision, (ICD-10) code for FH diagnosis was recently approved in October 2016 [15]. Diagnostic criteria for FH are the Simon Broome Register Criteria (SB), Dutch Lipid Clinical Network (DLCN), the US Make Early Diagnosis to Prevent Early Deaths Criteria (MEDPED), and the Japanese FH Management Criteria (JFHMC) [1,2,3,16]. In 2013, the European Atherosclerosis Society (EAS) published a consensus statement to set standard clinical diagnostic criteria in Europe [17]. More recently, a concordance analysis conducted among the Canadian population to produce Canadian standard diagnostic criteria showed strong agreement with the SB and DLCN criteria (κ = 0.969 and 0.966, respectively) [18]. The Modified System of Simplified Chinese Criteria also showed strong agreement with SB and DLCN criteria (*κ* = 0.993 and 0.958, respectively) and reported high sensitivity (91.9% and 100%) and high specificity (100% and 99.9%) [19]. Reports have shown that FH genetic testing can identify a causal gene variation in 60% to 80% of clinically-suspected FH cases and that large-scale DNA sequencing can identify FH cases that were either not clinically detected or potentially missed using an algorithmic approach [20,21]. Among these diagnostic criteria, the Canadian diagnosis guidelines, Modified System of Simplified Chinese Criteria, SB, and DLCN consider genetic testing to confirm FH diagnosis.

FH genetic testing is recommended because the results have the potential to change or influence patient management and identify at-risk first-degree, biological relatives [22]. In 2018, the American College of Cardiology reported (based on perspectives from cardiologists, primary care providers, and cardiovascular team members) that one of the key issues affecting the management of FH patients is the lack of education on guidelines for FH diagnosis, management, and treatment [23]. Few literature reviews have summarized evidence on the utility of FH genetic testing to address this education gap. Also, there is a dearth of knowledge about how FH testing is used in research and clinical settings globally and the overall value of FH genetic testing in terms of improving clinical outcomes [24,25,26]. Our review addresses these knowledge gaps, as we highlight research involving FH genetic testing and cascade screening to summarize available evidence on the utility of testing across diverse populations. We anticipate that this summary will be useful to inform clinical decision-making, insurance policies and coverage decisions for FH genetic testing, policy recommendations to reduce the clinical and public health burden of FH, clinical practice and guidelines to improve the management of FH populations, and ongoing research involving FH genetic testing.

## 2. Methods

### 2.1. Search Strategy

Full original research article reports were searched in PubMed/MEDLINE, EMBASE, and Yale University’s TRIP Medical Database, and Google Scholar in February of 2020 using Boolean string with MeSH terms, Diagnosis AND “Genetic Testing” AND “Hyperlipoproteinemia Type II.” Given that evidence on the utility of FH genetic testing spiked after 2016, based on our observations of “results by year” from a PubMed search, filters were used to find studies published after 2016 to ensure that the findings were not only relevant but timely. We carefully selected and reviewed studies reporting clinical and non-clinical outcomes following FH genetic testing in humans. Studies that did not report at least one clinical or non-clinical outcome were excluded. Original research articles were excluded if they were not published in the English language and if they were case reports (*n* = 1). Titles and abstracts were screened by one investigator (RHS). 

### 2.2. Data Extraction

We manually extracted key data on each study’s characteristics and clinical and non-clinical outcomes. Key characteristics extracted were author(s), number of participants screened, study participants’ age groups (pediatric, adult), geographic location, study participants’ ethnicities, FH diagnosis criteria used, clinical diagnosis, and reported phenotypes. We also extracted clinical and non-clinical outcomes, including patient reported outcomes (PROs) that followed genetic testing. Lastly, we determined, among studies meeting our inclusion criteria, if cascade screening was used to recruit family members for FH genetic testing, following the identification of probands/index cases. 

## 3. Results

**Figure 1:**
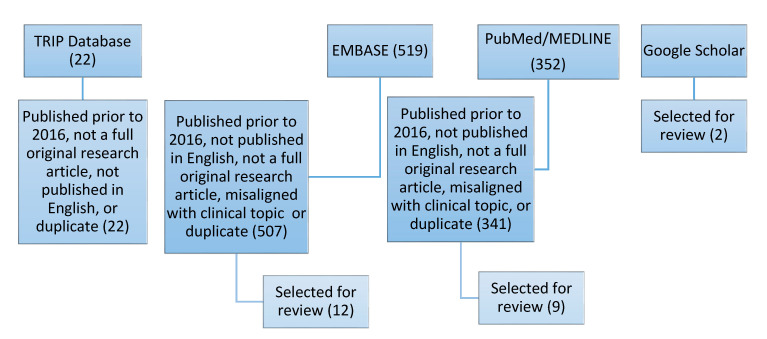
Literature Search Strategy and Results.

A total of 352 articles were retrieved from a search in PubMed/MEDLINE. After screening against the exclusion criteria, nine articles were selected for review. A search in EMBASE returned 519 results; however, after applying our inclusion/exclusion criteria and removing duplicates, 10 articles were selected for review. A TRIP Medical Database search returned zero results after screening against the inclusion/exclusion criteria. Two articles were found through a search in Google Scholar. A total of 21 articles were reviewed. Table A1 in the Appendix A summarizes key data extracted and clinical outcomes and non-clinical outcomes are summarized in Table A2 and Table A3 in Appendix A, respectively. Figure 1 presents our search strategy and the number of articles retrieved and selected for review.

Across these 21 studies, FH genetic testing was performed among a total of 60,893 clinically suspected adult and pediatric individuals across 11 countries in North and South America (83.40%), Europe (14.58%), Asia (1.55%), and Australia (0.45%). Studies largely reflect individuals tested in Italy, the US (Pennsylvania only), and Estonia (91.3% combined), and predominantly Caucasian populations. Clinically suspected cases presented across all 21 studies consisted of largely adult-only populations (*n* = 56,332 or 92.5% of total clinically suspected cases). The most represented continents include North America (mainly a US population in Pennsylvania), Europe, and Asia, although actual population ethnicities were rarely reported. DLCN was the most common clinical diagnostic criteria used (14 studies). FH phenotypes reported were LDL cholesterol > 130 mg/dL (or 3.4 mmoL/L), total blood cholesterol > 200 mg/dL (or 5.2 mmoL/L), xanthoma, xanthelasma, corneal arcus, and overt CAD (personal and individual). Five clinical outcomes following FH genetic testing were described or reported: improved FH diagnosis, treatment initiation or continued treatment, treatment modification, and improved total or LDL cholesterol levels. Only eight studies reported patient education on lifestyle management and genetic counseling following FH genetic testing, which we considered as non-clinical outcomes.

Although these 21 studies reported key clinical and non-clinical outcomes following FH genetic testing, PROs were reported in only two studies (Pang et al. and Jones et al.) and were: (1) concerns about receiving genetic test results and (2) out-of-pocket costs associated with FH genetic testing. PRO reporting was not consistent across these two studies; Pang et al. explored reasons why some parents in their study declined genetic testing for their children, citing parent concerns about possible stigmatization of genetic testing, while Jones et al. conducted qualitative interviews with seven patients to understand the patients’ overall experiences in receiving FH genetic test results [27,28]. 

Cascade screening was performed in 12 of the 21 studies, which occurred in Italy, Spain, Slovak Republic, India, Estonia, China, Vietnam, and Western Australia [28,29,30,31,32,33,34,35,36,37,38,39]. Among those 12 studies, the evidence suggested that first-degree relatives showed improved FH diagnosis and underwent treatment initiation, continued treatment, and/or treatment modification that resulted in improved LDL and total blood cholesterol. 

## 4. Discussion

FH genetic testing is not a new concept to FH diagnosis; however, its utility to address therapeutic challenges concerning FH have been unclear. The utility of genetic testing in any context is greater when genetic test results are actionable in that testing can inform better treatment decisions along with clinician and patient decision-making [40]. Here we provide broader clarity on this clinical topic by presenting and highlighting recent evidence showing that FH genetic testing has led to improved FH diagnosis, clinical outcomes (treatment initiation or continued treatment, improved LDL cholesterol or total blood cholesterol), and actionable outcomes (lifestyle management and genetic counseling) among diverse populations globally. Considering this evidence, FH genetic testing is recommended to confirm FH diagnosis or variant carrier status in clinically-suspected cases, especially cases involving very early-onset CAD where clinical signs of FH are less overt [31]. 

Our findings are consistent with a recent systematic review and meta-analysis showing that FH genetic testing can confirm FH diagnosis over clinical criteria alone, depending on the diagnostic algorithm and the method of analysis [41]. However, given that eight of the 21 studies reviewed reported only two non-clinical outcomes (education on lifestyle management and genetic counseling) following FH genetic testing, with no particular consistency in reporting these outcomes, there is opportunity to determine broader, non-clinical outcomes following FH genetic testing (e.g., behavioral outcomes like statin treatment adherence). 

Lipid levels in individuals can vary based on several factors (e.g. ethnicity, geography, etc.), thus rendering clinical diagnostic criteria unsuitable in some populations of FH variant carriers [42]. In fact, racial disparities in age of FH diagnosis have been reported among those registered within the International FH Foundation’s CASCADE Registry [43]. One study showed that among 3537 adults diagnosed with FH across 26 sites in the US, Asians received FH diagnosis at youngest ages (37 years [29,44]) and Blacks received FH diagnosis at latest ages (54 years [37,45]; *p* < 0.0001), placing the Black population at a possibly higher risk of premature cardiovascular disease due to undiagnosed FH [43]. Therefore, we assessed race and/or ethnicity reported across all 21 studies.

We also examined age characteristics across all 21 studies (adult versus pediatric (≤18 years), or both) and found that the study populations consisted of mostly adult populations (92.5% of total clinically suspected cases that underwent FH genetic testing). This finding shows that pediatric populations are largely absent from such investigations. Therefore, future research should examine clinical and non-clinical outcomes following FH genetic testing in children globally. 

Cascade screening is a robust, cyclical, and family tracing and screening mechanism that is used to identify an index/proband case and family members at risk for a genetic condition like FH [29]. In slightly more than half of the studies included in our review (12/21), cascade screening was used to identify patterns of FH risk and symptoms across first- and second-degree family members at various ages. Assessment of these patterns helped the clinicians diagnose and treat not just proband/index cases, but also family members with FH, thus showing early evidence on the clinical utility of FH genetic testing in cascade screening programs. Barriers to cascade screening for FH, however, must be overcome, which have been reported as challenges in identifying index/proband cases, suboptimal communication between the probands and family members, and geographic barriers to obtaining genetic testing services [20,44,46].

Race and geographic ethnicity/ancestry can provide clues about a person’s or population’s lifestyle, culture, and exposome, making race and/or ethnicity possible determinants of health outcomes seen among populations that share a specific genotype, phenotype, and/or culture. Studies presented in our review largely reflect cases in Italy, the US (Pennsylvania only), and Estonia (91.3% combined), and Caucasian populations. It is unlikely, however, that these cases reflect the general global FH population. Also, given that the two studies conducted within the US in the state of Pennsylvania occurred within the same health system, it is possible that the population reflected in Jones et al.’s study overlaps with the population reflected in Abul-Husn et al.’s study. Therefore, we recommend that future studies ascertain such outcomes in more geographically, ethnically, and health system diverse populations, especially since geography and ethnicity can be predictors of age of FH diagnosis, which might affect clinical outcomes and management [43].

Understanding PROs following FH genetic testing is important, as expert groups including the American College of Cardiology recommend that FH genetic testing should be offered to individuals of any age in whom a strong clinical index of suspicion for FH exists based on the examination of the patient’s clinical and/or family histories [47]. PROs can help clinicians and researchers understand and document patients’ preferences, complaints, and/or opinions following an intervention [48]. PROs were underreported in our review of studies related to FH genetic testing. The two studies that reported PROs showed that both patients and parents of patients have concerns about FH genetic testing and that FH genetic testing might be necessary to substantiate coverage for specialty medication. Therefore, future research should explore and report PROs to understand the humanistic aspects and economic implications of FH genetic testing. One recent case study highlights the importance of PROs; in one patient, FH genetic testing was required after it became clear that the patient responded poorly to various statin therapies and after the patient’s insurer denied coverage for a PCSK9 inhibitor [49]. Thus, future PRO studies can describe how FH genetic testing may or may not lead to better treatment with stronger statin therapies or PCSK9 inhibitors. 

Clinical guidelines for FH genetic testing have been established by the National Lipid Association (NLA) in 2011, the International FH Foundation in 2014, and the American Association of Clinical Endocrinologists and American College of Endocrinology (AACE/ACE) in 2017 [50]. These guidelines, however, might be outdated given the rapid advances of genomic science and medicine. Indeed, the basis of these guidelines does not include the evidence that we present here [2,51,52,53]. Additionally, in 2013, the EAS recommended FH genetic testing in cases where FH is probable or definite based on standard clinical diagnostic criteria, which mirror those of a 2018 International FH Foundation and American College of Cardiology expert consensus panel [17,47]. This expert consensus panel decision, however, cited only two studies included in our review (Amor-Salamanca et al. and Abul-Husn et al.). These considerations are important because diagnosis and treatment decisions made by clinicians and insurers usually align with published guidelines. Outdated guidelines without sufficient consideration of the clinical place of FH genetic testing can negatively affect FH patient and family diagnosis, management, and care [49].

Studies in other global regions not covered in this review are underway or beginning to examine the clinical and actionable outcomes discussed herein, to ascertain the clinical and public health utility of FH genetic testing. For example, the gulf familial hypercholesterolemia registry study is occurring among an adult population within the Arabian Gulf region where the prevalence of probable and definite FH (per DLCN criteria) is 1/232 [54]. Such studies will contribute to the rich and growing body of evidence showing how FH genetic testing might lead to overall favorable public health outcomes that could help address the current and growing global epidemic of cardiovascular disease. 

On the other hand, some studies have sought to determine when FH genetic testing is unnecessary. For example, as part of a national plan to make FH genetic testing available to all lipid clinics in England, English researchers sought to determine a triglyceride (TG) level at which FH is unlikely [55]. They determined among an adult population (*n* = 462, mean age of 51 years) that a TG level of 4 mmol/L showed a negative predictive value for FH at 100% (*p* < 0.0001) [55]. The researchers explained that such clinical questions are not answered in current clinical guidelines, yet are necessary to prevent unnecessary FH genetic testing under health policy programs that make FH testing widely available [55]. Research to determine the clinical threshold for FH genetic testing are also underway in other regions of the United Kingdom; the Wales FH service recently developed criteria for selecting patients for FH genetic testing by modifying DLCN scoring criteria and determined a score of ≥6 to be the proper threshold to offer FH genetic testing to the Welsh population [56]. In addition, the The BELgium Familial Hypercholesterolaemia Strategy (BEL-FaHST) recently indicated that genetic testing is not recommended in cases of definite FH per DLCN criteria (if >8) [57]. 

There are limitations to our literature search and review. First, the literature reviewed was searched and identified by a single reviewer (RHS) without comparison with a second reviewer. Therefore, the evidence summarized should be confirmed via discussion with the authors of the articles presented or by reviewing the articles directly. Further, the research articles presented herein were not reviewed for methodological quality before we summarized the evidence of clinical utility. Nonetheless, our review provides an important summary of the latest evidence on the utility of FH genetic testing. This review can be useful alongside other literature examining barriers to FH genetic testing and can be used to guide global calls to action, such as the Global Call to Action on Familial Hypercholesterolemia, and public policy recommendations to reduce the clinical and public health burden of FH [58,59]. 

## 5. Conclusions

Clinicians and insurers should consider this summary of evidence when determining (1) knowledge gaps on the patient-reported outcomes following FH genetic testing, evidenced by an unclear picture or inconsistent reporting of non-clinical outcomes following FH genetic testing; (2) if FH genetic testing may be appropriate in clinically suspected cases and how PROs are or can be used to understand what is needed to reach desired treatment outcomes; and (3) the clinical utility of FH genetic testing from a global perspective and across certain ethnic and age populations. Forthcoming clinical guidelines should also be based on the latest evidence herein. Finally, more research is needed to examine and determine the role of non-clinical outcomes and PROs following FH genetic testing, identify resources needed to inform treatment goals and help improve treatment outcomes in FH patients, and assess clinical outcomes following FH genetic testing in non-Caucasian and pediatric populations in the US and abroad. 

## Appendix A. Summary of Results

**Table A1:** Summary of Geographic, Age, and Clinical Characteristics: Familial Hypercholesterolemia (FH) Patient Populations Who Received Genetic Testing.

Citation	Geographic Location/Ethnicity	Age Characteristics (Adult, Pediatric)	Percent of Clinically Suspected Individuals Tested (*n* = 60,893)	Clinical Diagnosis (Diagnostic Criteria, Number Diagnosed Before or in Lieu of Genetic Testing)	Phenotype(s) Reported
Vohnout et al. [29]	Slovak Republic/Not reported	Adult	0.01	Possible FH (SB, *n* = 3)	Total blood cholesterol > 200 mg/dL or 5.2 mmol/L LDL cholesterol > 130 mg/dL or 3.4 mmol/L
Sperlongano et al. [30]	Italy/Not reported	Adult	0.01	Definite FH (DLCN, *n* = 4) Probable FH (DLCN, *n* = 3)	LDL cholesterol > 130 mg/dL or 3.4 mmol/L Total blood cholesterol > 200 mg/dL or 5.2 mmol/L Xanthoma Corneal arcus Overt CAD (personal and family history)
Amor-Salamanca et al. [31]	Spain/88.3% Caucasian	Adult	0.17	Definite FH (DLCN, *n* = 12; SB, *n* = 2) Probable FH (DLCN, *n* = 16) Possible FH (DLCN, *n* = 52; SB, *n* = 26) Unlikely FH (DLCN, *n* = 23; SB, *n* = 75)	LDL cholesterol > 130 mg/dL or 3.4 mmol/L Total blood cholesterol > 200 mg/dL or 5.2 mmol/L Overt CAD (personal)
Setia et al. [32]	India/Asian	Adult Pediatric	0.22	Not reported	LDL cholesterol > 130 mg/dL or 3.4 mmol/L Total blood cholesterol > 200 mg/dL or 5.2 mmol/L Xanthoma Xanthelasma Corneal arcus Overt CAD (family history)
Pang et al. [28]	Western Australia/Not reported	Adult Pediatric	0.45	Not reported	LDL cholesterol > 130 mg/dL or 3.4 mmol/L Total blood cholesterol > 200 mg/dL or 5.2 mmol/L
Averna et al. [60]	Italy/Not reported	Adult Pediatric (Note: the number receiving genetic testing in each group was unspecified)	5.59	Definite FH (DLCN, n-1131) Probable FH (DLCN, *n* = 821)	LDL cholesterol > 130 mg/dL or 3.4 mmol/L Xanthoma Corneal arcus Overt CAD (personal and family history)
Minicocci et al. [61]	Italy/Not reported	Pediatric	0.13	Definite or Probable FH (DLCN, *n* = 64) Definite FH (SB, *n* = 0) Definite FH (EAS, *n* = 49)	Total blood cholesterol > 200 mg/dL or 5.2 mmol/L LDL cholesterol > 130 mg/dL or 3.4 mmol/L Overt CAD (personal and family history)
Séguro et al. [62]	France/Not reported	Adult Pediatric	0.56	Definite or Probable FH (DLCN, *n* = 344)	LDL cholesterol > 130 mg/dL or 3.4 mmol/L Total blood cholesterol > 200 mg/dL or 5.2 mmol/L Xanthoma Xanthelasma Corneal arcus Overt CAD (personal and family history)
Abul-Husn et al. [21]	Pennsylvania, US/98.4% Caucasian	Adult	83.30	Definite FH (DLCN, US MEDPED, *n* = 53) Probable FH (DLCN, US MEDPED, *n* = 497) Possible FH (DLCN, US MEDPED, *n* = 5465) Unlikely FH (DLCN, US MEDPED, *n* = 40,270)	LDL cholesterol > 130 mg/dL or 3.4 mmol/L Overt CAD (personal)
Jones et al. [27]	Pennsylvania, US/100% Caucasian	Adult	0.04	Not reported	LDL cholesterol > 130 mg/dL or 3.4 mmol/L Overt CAD (personal)
Wang et al. [63]	China/Not reported	Adult Pediatric	0.02	Definite FH (DLCN, *n* = 5)	LDL cholesterol > 130 mg/dL or 3.4 mmol/L Total blood cholesterol > 200 mg/dL or 5.2 mmol/L Xanthoma Corneal arcus
Wu et al. [33]	China/Not reported	Adult Pediatric	0.31	Not reported	LDL cholesterol > 130 mg/dL or 3.4 mmol/L Total blood cholesterol > 200 mg/dL or 5.2 mmol/L Xanthoma Corneal arcus Overt CAD (personal and family history)
Truong et al. [34]	Vietnam/Not reported	Adult Pediatric	0.15	Likely FH (Starr et al. 2008 method, *n* = 9) Unlikely FH (Starr et al. 2008 method, *n* = 9)	LDL cholesterol > 130 mg/dL or 3.4 mmol/L Total blood cholesterol > 200 mg/dL or 5.2 mmol/L Xanthoma Xanthelasma Corneal arcus Overt CAD (personal and family history)
Tan et al. [35]	China/Not reported	Adult	0.15	Not reported (DLCN, n not reported)	LDL cholesterol > 130 mg/dL or 3.4 mmol/L Total blood cholesterol > 200 mg/dL or 5.2 mmol/L Xanthoma Xanthelasma Overt CAD (personal)
Gómez et al. [64]	Argentina/European ancestry (predominant) and Native American and African ancestry (lower proportion)	Adult	0.06	Definite FH (DLCN, *n* = 38)	LDL cholesterol > 130 mg/dL or 3.4 mmol/L Overt CAD (personal)
Rubio-Marín et al. [36]	Spain/Not reported	Adult Pediatric	0.18	Probable FH (DLCN, *n* = 132)	LDL cholesterol > 130 mg/dL or 3.4 mmol/L Total blood cholesterol > 200 mg/dL or 5.2 mmol/L Xanthoma Corneal arcus Overt CAD (personal and family history)
Cui et al. [42]	China/Not reported	Adult	0.37	Definite or Probable FH (DLCN, *n* = 12) Definite or Probable FH (modified DLCN, *n* = 49)	LDL cholesterol > 130 mg/dL or 3.4 mmol/L Total blood cholesterol > 200 mg/dL or 5.2 mmol/L
Ibarretxe et al. [37]	Spain/Not reported	Adult Pediatric	0.09	Definite FH (DLCN, *n* = 76)	LDL cholesterol > 130 mg/dL or 3.4 mmol/L Total blood cholesterol > 200 mg/dL or 5.2 mmol/L
Chan et al. [38]	China/Not reported	Adult	0.16	Definite FH (DLCN, *n* = 38) Probable FH (DLCN, *n* = 34) Possible FH (DLCN, *n* = 24)	LDL cholesterol > 130 mg/dL or 3.4 mmol/L Total blood cholesterol > 200 mg/dL or 5.2 mmol/L Xanthoma Xanthelasma Corneal arcus Overt CAD (personal and individual)
Cao et al. [65]	China/Not reported	Adult	0.17	Definite FH (DLCN, *n* = 16; SB, *n* = 10) Probable FH (DLCN, *n* = 12) Possible FH (DLCN, *n* = 49; SB, *n* = 8) Unlikely FH (DLCN, *n* = 28; SB, *n* = 87)	LDL cholesterol > 130 mg/dL or 3.4 mmol/L Total blood cholesterol > 200 mg/dL or 5.2 mmol/L Xanthoma Overt CAD (personal individual)
Alver et al. [39]	Estonia/Not reported	Adult	7.84	Not reported	LDL cholesterol > 130 mg/dL or 3.4 mmol/L

Acronym Key: SB = Simon Broome, DLCN *=* Dutch Lipid Clinical Network, MEDPED = Make Early Diagnosis to Prevent Early Deaths System, EAS = European Atherosclerosis Society, CAD = Coronary artery disease.

**Table A2:** Summary of Clinical Outcomes Reported Per Study.

Clinical Outcome(s) Reported Among Populations Tested	Percent of Total Clinically Suspected Individuals Tested (*n* = 60,893)	Citation
Treatment initiation	92.68	Vohnout et al. [29] Sperlongano et al. [30] Setia et al. [32] Pang et al. [28] Abul-Husn et al. [21] Jones et al. [27] Wang et al. [63] Wu et al. [33] Truong et al. [34] Rubio-Marín et al. [36] Alver et al. [39]
Continued treatment	92.26	Amor-Salamanca et al. [31] Setia et al. [32] Pang et al. [28] Abul-Husn et al. [21] Jones et al. [27] Gómez et al. [64] Rubio-Marín et al. [36] Alver et al. [39]
Modified treatment and/or dose	8.06	Jones et al. [27] Rubio-Marín et al. [36] Alver et al. [39]
Improved LDL cholesterol	84.00	Sperlongano et al. [30] Pang et al. [28] Abul-Husn et al. [21] Jones et al. [27] Wang et al. [63] Rubio-Marín et al. [36]
Improved total blood cholesterol levels	0.01	Sperlongano et al. [30]

**Table A3:** Summary of Percent of Individuals Tested and Non-Clinical Outcomes Reported Following FH Genetic Testing.

Non-Clinical Outcome(s) Reported Among Populations Tested	Percent of Total Clinically Suspected Individuals Tested (*n* = 60,893)	Author/Study
Education on lifestyle management	14.60	Vohnout et al. [29] Setia et al. [32] Pang et al. [28] Averna et al. [60] Wu et al. [33] Rubio-Marín et al. [36] Alver et al. [39]
Genetic counseling	5.63	Averna et al. [60] Jones et al. [27]
None reported	86.33	Sperlongano et al. [30] Amor-Salamanca et al. [31] Minicocci et al. [61] Séguro et al. [62] Abul-Husn et al. [21] Wang et al. [63] Truong et al. [34] Tan et al. [35] Gómez et al. [64] Cui et al. [42] Ibarretxe et al. [37] Chan et al. [38] Cao et al. [65]
